# The Bet-Hedging Strategies for Seedling Emergence of *Calligonum mongolicum* to Adapt to the Extreme Desert Environments in Northwestern China

**DOI:** 10.3389/fpls.2018.01167

**Published:** 2018-08-08

**Authors:** Baoli Fan, Yongfeng Zhou, Quanlin Ma, Qiushi Yu, Changming Zhao, Kun Sun

**Affiliations:** ^1^College of Life Science, Northwest Normal University, Lanzhou, China; ^2^State Key Laboratory of Desertification and Aeolian Sand Disaster Combating, Gansu Desert Control Research Institute, Lanzhou, China; ^3^State Key Laboratory of Grassland Agro-Ecosystems, School of Life Sciences, Lanzhou University, Lanzhou, China; ^4^Department of Ecology and Evolutionary Biology, University of California, Irvine, Irvine, CA, United States

**Keywords:** desert pioneer shrub, seed burial depth, light intensity, seed age, seedling emergence strategy

## Abstract

*Calligonum mongolicum* is a dominant native perennial shrub on sand dunes in arid deserts of northwestern China, and is therefore widely used in sand dune stabilization in these regions. However, it remains largely unknown how seedling emergence of *C. mongolicum* has adapted to unpredictable sand movement and extreme drought. Here we examined effects of seed burial depth, light intensity, and seed age on seedling emergence, and considered seed germination and seedling emergence strategies for the shrub’s adaption to the desert environment. In our pot experiment, the optimum seeding depth for emergence of *C. mongolicum* was 2 cm, indicating that for germination and seedling emergence only moderate sand burial is required. Light intensity at the surface soil (0 cm) was important for seedling emergence, while there was no significant difference between 50 and 20% light flux density, at burial depths of 1 and 2 cm, indicating that *C. mongolicum* seeds had adapted to sand burial, while not exposure from sand erosion. We also found *C. mongolicum* seedlings emerged in spring and in late summer to early autumn. Meanwhile, seedling emergence percentage for 3-year-old seeds was similar to that of 1-year-old seeds, which meant that *C. mongolicum* seeds were well preserved under normal sand dune conditions, thus were capable of developing a persistent, but shallow soil seed-bank. These results indicated that germination and seedling emergence take a bet-hedging strategies to adapt to variable desert environments. Our study confirmed that *C. mongolicum* desert shrubs combine strategies in its adaption to arid and variable sand environments.

## Introduction

Understanding how organisms cope with and adapt to changes in their environments is a central theme to evolutionary ecology (Botero et al., 2015). Germination and seedling emergence are critical transition periods during which a plant leaves the relative safety of the seed stage and enters the highly vulnerable seedling stage (Gremer et al., 2016). The ecology of seeds and their germination patterns determine a species’ adaptation to various environments and allows us to explain and predict ecological dynamics (Huang et al., 2016). In deserts, seeds and plants often face varying degrees of sand burial and exposure by wind erosion, thus their ability to respond to these environmental cues is of great importance for successful seed germination, seedling emergence, and initial establishment, which may ultimately affect population viability. It is well known that seed germination and seedling emergence depend on many factors, such as soil light intensity (Tobe et al., 2006; Zhang et al., 2014), moisture (Tobe et al., 2005), temperature (Liu et al., 2013) and others. Sand burial and exposure from wind erosion can easily affect these factors in the microenvironment of dune plants (Poulson, 1999; Qu et al., 2014), which may influence seed germination and seedling emergence of the plants. Although tolerance to burial was found to differ considerably among species, burial was considered an important selective force (Maun, 2004; Dech and Maun, 2005; Liu et al., 2007; Xu et al., 2013).

For some species, the timing of emergence plays a critical role in seedling establishment (Bush and Van Auken, 1991) and as expected, also a strong selective force (Gremer et al., 2016). Delaying germination for a year or within season time scales, which is described as biological bet-hedging, has been demonstrated to significantly benefit plants by escaping unfavorable conditions and spreading risk of seedling failure (Simons, 2009; Gremer and Venable, 2014; Gremer et al., 2016). Meanwhile, the volume of seeds persisting in the soil reflects the relative risks around a seed remaining viable in the soil and to germinate under conditions that are more favorable. Plants that can reduce the effect of environmental uncertainty in this way are considered to exhibit a bet-hedging strategy (Arroyo et al., 2006; Yu et al., 2007). Many studies have reported on seed germination and seedling emergence of annual plants growing in different deserts of the world (Went, 1949; Tevis, 1958; Venable and Brown, 1988; Venable, 2007; Gremer et al., 2016; Huang et al., 2016). However, very few studies of this type have been applied to desert perennial shrubs (Baskin et al., 1993; Gutterman, 2000). The reproductive strategies employed by plants are very important for adaption to extreme desert environments. Accordingly, it is either phenotypic plasticity (Angert et al., 2010; Botero et al., 2015) or a bet-hedging strategy (Simons, 2011) or both (Simons, 2014; Gremer et al., 2016) that are essential for plants to adapt to desert environments. Thus, it remains unknown which strategy was adopted by pioneer shrubs to inhabit mobile sand dunes and to adapt to unpredictable sand movement and extreme drought. *Calligonum mongolicum* (Polygonaceae) is a dominant native perennial shrub in active sand dunes in arid deserts of northern China, and is widely used for vegetation restoration in desert region (Fan et al., 2018). In these sandy habitats, each mature shrub produces numerous quantities of fruit and seeds are dispersed considerable distances by prevailing winds. In recent years, *C. mongolicum* exhibited a population expansion in mobile sand dunes in the arid Minqin Desert. Previous greenhouse experiments on the species have shown the impact of hydration–dehydration cycles as well as different pre-sowing seed treatments on seed germination of several *Calligonum* sp. (Ren and Tao, 2003, 2004), despite all of the above, it remains unclear how *C. mongolicum* seedling emergence has adapted to the sand environment. In this study, we investigated *C. mongolicum* seedling emergence by examining the effects of seed burial depth, light intensity and seed age. The objective was to investigate seed germination and seedling emergence strategies in the shrub’s adaption to arid desert environments, and to evaluate its usage in conservation strategies of mobile sand dunes.

## Materials and Methods

### Seed Collection and Selection

Over the previous three growing seasons in the Minqin County, fresh seeds from *C. mongolicum* were collected from the surface of desert sand dunes under the shrubs. These seeds were air dried in paper bags and stored in year-collected batches for use in germination and emergence pot trials. Meanwhile, we randomly selected 3 subsamples of 100 seeds from these fresh seeds and measured, seed length, seed width, seed bristle length and weight. All seeds were air-dried at room temperature by spreading on tables. Prior to the pot experiment, 50 seeds from each seed age group (one- and three-year-old), were subjected to the tetrazolium chloride viability test, which indicated that in each seed age group >90% of the embryos appeared light colored and thus were regarded as viable.

### Field Seedling Emergence Experiments

Based on *C. mongolicum*’s ability to endure considerable sand movement, the potted seedling emergence experiment included three treatments: sand burial, burial depth with light intensity, and burial depth with seed age.

### Sand Burial Experiments

All sand burial treatments consisted of five replicates of 20 seeds. Each replicate of 20 seeds were planted at 0, 1, 2, 4, 6, and 8 cm depths in plastic pots (14 cm in diameter) filled with sand from mobile dunes. The potted sand dune soil dried quickly in the hot, dry conditions of our study site, however, previous unpublished work indicated that flooding with excess irrigation water limited *C. mongolicum*’s emergence. Therefore, the pots were moistened daily with a garden watering-can, fitted with a fine rosette to mimic light rainfall in very small quantities, as would be the case in the desert, i.e., moist soil with maximized air filled porosity, the volume of which was based on our previous unpublished work. Meanwhile, seedling appearance at the sand surface was recorded daily for 5 months, and on completion of the study.

### Light Intensity, Seed Age, and Sand Burial Experiments

Seed burial depth preliminary experiment on seed burial depth indicated an optimal seeding depth of 2 cm, followed by 1 cm and with no germination from seeds placed on the soil surface (0 cm). Therefore, these three depths (0, 1, and 2 cm) were chosen to assess emergence response to light density and burial depth. Shade netting was used to obtain three levels of light intensity, 100% natural light (CK), 50% light flux density (LFD) and 20% LFD.

To assess the impact of seed age on germination percentage, seeds that were dry stored for 1 and 3 years were sown in pots containing sandy soil from mobile sand dunes at five seed burial depths (0, 1, 2, 4, and 6 cm). Since seedlings failed to emerge from 3-year-old seeds at burial depths of 0 and 6 cm from previous work, time to emergence was only recorded at 1, 2, and 4 cm seeding depths for both seed ages.

### Statistical Analyses

Seedling emergence was measured using two indices: the final emergence percentage and the initial emergence time. The percentage of seedling emergence was the number of seedlings at the end of the experiment divided by the number sown. The initial emergence time was defined as the time from sowing to the first seedling emergence day, i.e., days after sowing (DAS).

The statistical package SPSS 16.0 software (SPSS, Chicago, IL, United States) was used for the analyses. One-way ANOVA was used to test the differences in initial emergence time and the final percentage emergence among different seed burial depths. A two-way ANOVA was used to analyze the effect of light intensity, seed burial depth and their interaction, seed age, seed burial depth, and their interaction on the seedling emergence percentage. Where there was a significant difference, a multiple comparison LSD determined the level of difference among treatments at *P* < 0.05. Before analysis, data were arcsine transformed for homogeneity of variance. Data means ± SE and figures were created with Origin 8.0.

## Results

Mean seed weight of the collected *C. mongolicum* seeds was 0.11 ± 0.00 g (mean ± SE) with a length of 1.64 ± 0.16 cm. Mean bristle length was 4.15 ± 0.91 mm and was about 25 percent of the length of the seed.

### *C. mongolicum* Seedling Emergence Process at Different Seeding Depths

**FIGURE 1:**
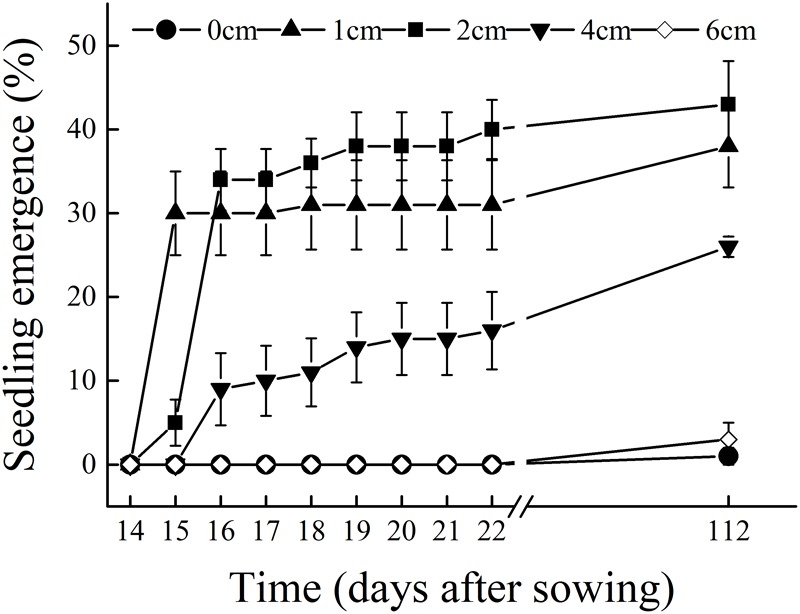
Seedling emergence process of *C. mongolicum* at different seed burial depths [14–22 days after sowing (DAS) is in May; 112 DAS is in August].

**FIGURE 2:**
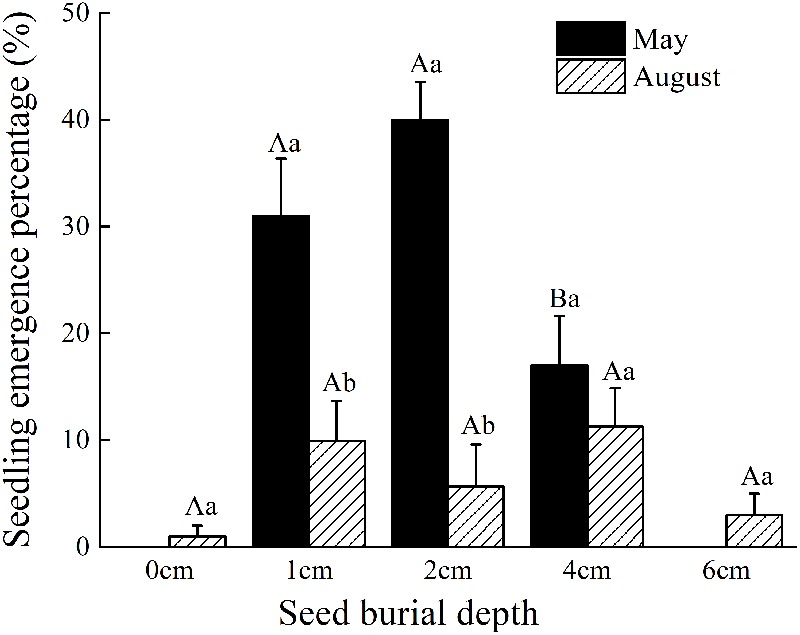
Effects of seed burial depth on the seedling emergence percentage of *C. mongolicum* in June and August. Values with different uppercase letters within each seed burial depth in the same month are significantly different (*P* < 0.05); Values with different lowercase letters between months under same seed burial depth are significantly different (*P* < 0.05).

Under field conditions and within the same season, *C. mongolicum* seedlings largely emerge at two different times during June and August. In the pot trials, seedlings at seed burial depths of 1 and 2 cm began to emerge 15 DAS, while those at 4 cm depth began to emerge 16 DAS. The DAS did not differ significantly among seed burial depths of 1, 2, and 4 cm (**Figure 1**). However, at 0, 6, and 8 cm seeding depths, seedlings failed to emerge in July, but finally emerged in August. Emergence percentage at 1 and 2 cm seeding depths was significantly higher than 0, 4, and 6 cm seeding depths. Seedling emergence percentage was only 1% at 0 cm and 3% at 6 cm seeding depths, in contrast to 7, 3, and 10% emergence for 1, 2, and 4 cm seeding depths in August, respectively (**Figure 2**). Seedling emergence in August at 1 and 2 cm depths was significantly lower than those at corresponding depths in July (**Figure 2**). Two-way ANOVA showed that the percentage of seedling emergence of *C. mongolicum* were significantly affected by soil depth, time and their interactions (*P* < 0.001) (**Table 1**).

**Table 1:** Effects of time and seed burial depth on the seedling emergence percentage.

Source	SS	*df*	MS	*F*
Time	1633.02	1	1633.01	29.72^∗∗∗^
Burial depth	4358.78	4	1089.69	19.83^∗∗∗^
Time × Burial depth	2532.52	4	633.13	11.52^∗∗∗^

*^∗∗∗^*P* < 0.001.*

### Effects of Light Intensity and Sand-Burial Depth on Seedling Emergence

**FIGURE 3:**
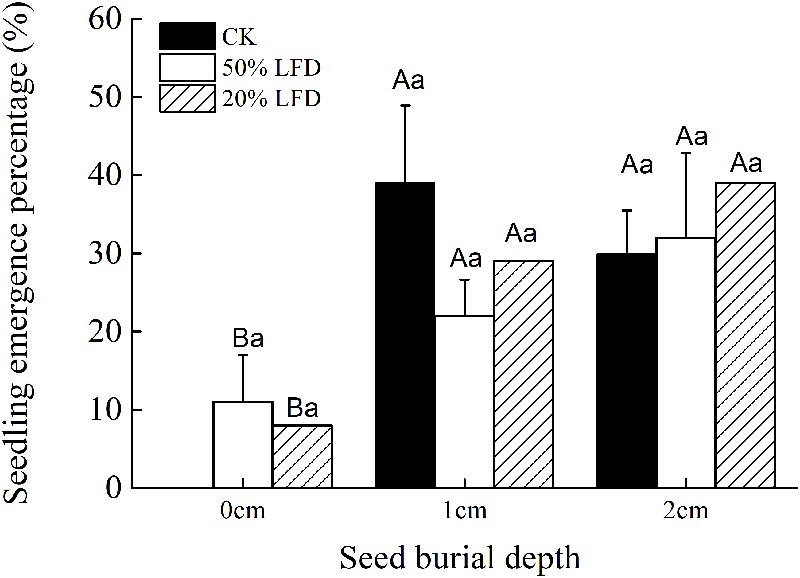
Effects of light intensity and seed burial on the seedling emergence percentage of *C. mongolicum* (Mean ± SE). Values with different uppercase letters within each light treatment under same seed burial depth are significantly different (*P* < 0.05); Values with different lowercase letters among each seed burial depth under same light are significantly different (*P* < 0.05).

Seeding depth had a significant effect on seedling emergence of *C. mongolicum*, while emergence was not affected by light intensity or the interaction of light intensity and seed burial depth (**Table 2**). Seedling emergence experiments under different light densities showed that seedlings failed to germinate placed on the sand surface (0 cm) in 100% LFD treatments, but emerged in 50 and 20% LFD with no significant difference between the two treatments, and both were not significantly different from that observed in 100% LFD (CK) (**Figure 3**). Meanwhile, there were no significant difference in seedling emergence percentages at 1 and 2 cm seeding depths among different light densities or different seed burial depths (**Figure 3**).

**Table 2:** Two-way analysis of variance of the effect of light intensity, seed burial depth and their interactions on final seedling emergence in pot trials.

Source	SS	*df*	MS	*F*-value	*p*-value
Light intensity	103.333	2	51.667	0.216	0.807
Burial depth	6603.333	2	3301.667	13.789	0
Light intensity **×** Burial depth	1173.333	4	293.333	1.225	0.317

### Effects of Seed Age and Sand-Burial Depth on Seedling Emergence

**FIGURE 4:**
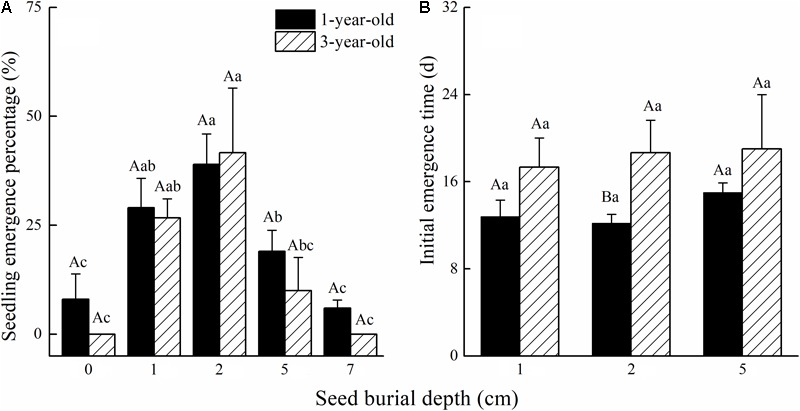
Effects of seed age and seed burial depth on **(A)** seedling emergence and **(B)** days of first emergence of *C. mongolicum* (*P* < 0.05).

*Calligonum mongolicum* seedling emergence in pot trials was not affected by seed age or the interaction of seed age and seed burial depth. However, seed burial depth had a significant influence on seedling emergence (**Table 3**). Seedling emergence from the 2 cm seed burial depth was 39% for 1-year-old and 41% for 3-year-old seed. Seedling emergence significantly improved at 4 cm seeding depth. Seed buried at 1 cm was similar in emergence to 2 cm seeding depth, but as burial depth increased to 4 cm, seedling emergence significantly decreased by 51.3% for 1-year-old seeds and 76% for 3-year-old seeds (**Figure 4A**). The initial seedling emergence time (DAS) was not significantly different between 1- and 3-year-old seed, at either 1 or 4 cm seeding depths, except at 2 cm seeding depth (**Figure 4B**).

**Table 3:** Two-way analysis of variance of the effect of seed age, seed burial depth and their interactions on final seedling emergence in pot trials.

Source	SS	*df*	MS	*F*-value	*p*-value
Seed age	46.944	1	46.944	0.204	0.657
Burial depth	2503.472	2	1251.736	5.438	0.014
Seed age × Burial depth	128.472	2	64.236	0.279	0.76

## Discussion

*Calligonum mongolicum* shrubs are simultaneously and quite often exposed to wind erosion and sand burial, but serve well as a pioneer species in the Minqin deserts (Fan et al., 2018). In this study, we investigated the effects of burial depth, seed age, and light intensity on the seedling emergence of *C. mongolicum*, and found that the species in seedling emergence stage are well adapted to sand, and have a procreation bet-hedging strategy to adapt to the extreme environment stresses in the desert.

### Adaptation to Sand Burial Environments

Improved growth vigor of individual plants after sand burial is a characteristic feature of dune perennials, well-adapted to frequent sand burial conditions (Maun, 1998). In this study, *C. mongolicum* seeds showed characteristics typical for a species well adapted to sand burial environments. On one hand, seeds buried by sand ranging from 1 to 6 cm burial depths, showed a much better emergence, while our unpublished data on seed germination of *C. mongolicum* in laboratory, showed almost no seed germinated in Petri dishes (2.00 ± 1.22%) under light conditions or (1.00 ± 1.00%) under dark conditions, respectively. This is due to the high degree of seed dormancy of *C. mongolicum* seeds, which contain water-soluble germination inhibiting substances in peels and seeds (Yu and Wang, 1998). In the desert, these inhibition substances could decrease or even disappear with time, due to high gaseous exchange in sandy soil, which increases seedling emergence by up to 40%. Meanwhile, Baskin and Baskin (1989) noted that a “primary reason of non-dormant seeds not to germinate in the soil, is not having the light requirement for germination.” Similarly, our study showed that light at surface soil (0 cm seed burial depth) was important for seedling emergence, while there was no significant difference between 50 and 20% LFD at burial depths of 1 and 2 cm, indicating that seeds of *C. mongolicum* may not require high-density light for their germination and emergence. Here we have extended this to sand burial, as the direct effect of burial on seed was a loss of illumination. A similar reaction to light had been reported in other species distributed in deserts, e.g., *Agriophyllum squarrosum* (Tobe et al., 2005), *Larrea divaricata* (Barbour, 1969), and *Citrullus colocynthis* (Koller et al., 1963). However, unpublished data of seed germination in Petri dishes under light and dark conditions were not significantly different (*F* = 0.4, *p* = 0.55), which may be due to the very low seed germination percentages for these two light treatments. The data also indicated that light may not be the decisive factor for *C. mongolicum* germination, since sand burial could change many micro-environmental factors simultaneously. In general, there was substantial evidence from our research to conclude that *C. mongolicum* seeds are well adapted to sand burial, while not sand erosion.

Although seedling emergence of *C. mongolicum* possess adaptations for sand burial, in our pot experiment, seedlings of *C. mongolicum* emerged from 1, 2, 4, and 6 cm seeding depths, but not from depths greater than 6 cm. The results are essentially in agreement with other reports on seed germination of desert species, which is directly related to the depth at which the seeds are buried (Baskin and Baskin, 1989; Zhang and Maun, 1990; Gutterman, 1993; Benvenuti et al., 2001; Zheng et al., 2005; Liu et al., 2011; Zhu et al., 2014; Luo and Zhao, 2015). Both oxygen deficiency and higher mechanical resistance may inhibit germination and seedling elongation in deeper sand (Tobe et al., 2005). Additionally, the optimum depth for improved seedling emergence of a species is generally acknowledge to be related to seed size. *C. mongolicum* seeds at about 1.42–1.84 cm of fruit length, and 1.1–1.93 cm fruit width, indicated that the optimum seeding depth for seedling emergence was near 2 cm, which is in line with a previous investigation in the same species (Ren et al., 2002). Based on this result, seedling emergence of *C. mongolicum* in sand is regulated by seed burial depth, and the vertical distribution of seed bank is expected to determine the proportion of seeds able to germinate.

### The Bet-Hedging Strategy

In a variable environment, organisms must have strategies to deal with unpredictable changes in conditions (Gremer et al., 2016). The bet-hedging strategy benefits plants by avoiding unfavorable conditions and to spread risks from extreme drought (Simons, 2009; Gremer and Venable, 2014; Gremer et al., 2016). Seed germination and seedling emergence timing influence the environmental conditions that seedlings will experience (Barga et al., 2017), thus they have important ecological implications (Huang et al., 2016). Under procreation bet-hedging, germinating and emerging at multiple times during the season can reduce the risk of emergence failure for seeds buried in deep sand (Simons, 2009). In Minqin desert, emerging and vulnerable seedlings are highly likely to suffer or die, due to erosion or sand burial from strong and unpredictable winds during early spring to summer. *C. mongolicum* seedling emergence generally occurs under suitable soil water and temperature conditions in spring. However, only about 40% of seedlings emerged under these conditions, while a further 7% emerged in the late summer or early autumn. Seeds that emerge in either July or August indicate that *C. mongolicum* emergence takes a “cautious” strategy to spread the risks, which is described as biological bet-hedging (Botero et al., 2015). The seedling emergence rate of *C. mongolicum* under pot trials in the field, was much higher at moderately seed burial depths in spring, than in early autumn, which could lower survival risk under more favorable conditions for growth and reproduction (Gremer et al., 2016), especially during annual summer rains in northwestern China.

Additionally, seeds that persist in the soil seed bank for more than 1 year are considered to exhibit a bet-hedging adaptation to environmental uncertainty (Yu et al., 2007). Plant communities in arid habitats persist in the face of high temperatures and low rainfall (Ooi et al., 2009). Thus only persistent long-lived seed banks will ensure that viable seeds are available to take advantage of sporadic rainfall events (Gutterman, 1993; Facelli et al., 2005) and play a significant role in the regeneration of plant communities (Bekker et al., 1997; Norbert and Annette, 2004). Our results indicated that final seedling emergence percentage for 3-year-old seeds was similar to that of 1-year-old seeds. As mentioned previously, mature *C. mongolicum* seeds contain significant germination inhibitors, resulting in deep dormancy (Yu and Wang, 1998). *C. mongolicum* seed viability appeared long-lived under natural conditions due to deep dormancy, which offers an effective mechanism for seeds to persist in the soil (Grime, 1981; Dalling et al., 2011). Thus, the results presented here suggest that *C. mongolicum* could be capable of developing a short-term shallow soil seed-bank in dunes, while waiting for suitable conditions to germinate, and it is thought to have evolved a bet-hedging adaptation in response to the unpredictable desert environment.

## Conclusion

The present study indicated that faced with variable winds and scarce precipitation in Minqin desert environment, *C. mongolicum* has adopted bet-hedging strategies to adapt to desert conditions. On one hand, the species has evolved to spread the risk in an uncertain environment through an extended germination time. On the other hand, it has developed a persistent soil seed-bank strategy to adapt to wind erosion and sand burial. Although this represents an interesting perspective on the adaptation of *C. mongolicum* to sand dune environments, we stress that more investigations on natural-regeneration are required to understand the reproductive ecology of this important pioneer perennial shrub.

## Author Contributions

BF conceived, designed, analyzed the data, and wrote the manuscript. BF, QM, and QY performed the filed study. YZ, KS, and CZ reviewed and supervised the manuscript. All authors contributed critically to the drafts and provided final approval for publication.

## Conflict of Interest Statement

The authors declare that the research was conducted in the absence of any commercial or financial relationships that could be construed as a potential conflict of interest.
